# Punctured Fractures of Cranium

**Published:** 1854-06

**Authors:** J. C. Hughes

**Affiliations:** Professor of Surgery in the Iowa Medical College


ERRATA.—In the second paragraph on this page, read *Fissure* for *Tissue.*

THE

### IOWA MEDICAL JOURNAL.

VOL. I. KEOKUK, IOWA, JUNE, 1854. NO. 11.

### ORIGINAL COMMUNICATIONS.

### *Punctured fractures of the Cranium.*

BY J. C. HUGHES, M. D.,

Professor of Surgery in the Iowa Medical College.

The varieties and extent of fractures in connection with the Cranium, are numerous, and demand almost an equal variety of treatment.

Simple Tissue, may exist without producing any alarming symptoms, but when accompanied by laceration or extravasation producing compression, inflammation and suppuration, it amounts to one of the most serious complications in connection with the brain, and demands of the Surgeon prompt and skillful interference. The safety of the patient depends more upon the preventive than curative treatment, and *per consequence,* the primary symptoms should be closely watched. If inflammatory symptoms present themselves, the most energetic treatment is demanded. When occurring in connection with the *base* of the *brain,* laceration of the dura mater and internal hemorrhage is almost always present, rendering life perilous, and surgical interference almost useless.

The most common form of fracture of the Cranium which we are called upon to treat, differs from mere fissure, and is somewhat analogous to the comminuted fracture of the long bones, having one or more fragments, and yet there may or may not be displacement, which has much to do in our favorable prognosis of the case. If no displacement occurs, there may be no more difficulty than in simple fissure and the case should be let alone, watching closely the symptoms. Should the external wound connect, converting it into a compound fracture, which is often the case, the primary symptoms may seem to be aggravated, but in the farther advancement of the case, when inflammation and suppuration have occurred, the external wounds often proves advantageous, by allowing accumulations to escape, thereby preventing injury to more important parts.

Should *displacement* in ordinary fracture exist, with symptoms of compression, the danger would be much greater, yet much depends upon the portion of brain affected; as it is well known that the upper and anterior part is much less liable to suffer from compression than would the base, or sides of its hemispheres. Here again we would urge the closest attention to symptoms, and when urgent, the most active treatment. But in these cases, the use of the trephine has been too strongly urged by the profession. It is valuable in treatment, but should be used with the greatest caution, not as has so often been done, recklessly and at the onset.

A question here arises; should we interfere with the trephine in all cases, even when depressed bone is present? Many of the profession would, and many writers upon the subject have answered in the affirmative. But experience has taught that in some cases, it would be hazardous. Our object in resorting to the operation would be to remove the cause of compression as well as the exciting cause of inflammation of the membranes of the brain. When the symptoms are very urgent, this course is demanded; but if even symptoms of compression are present, and these symptoms are declining rather than becoming more aggravated, we would prefer not to operate, expecting that the brain would accommodate itself to the depressed bone, a condition which would be followed by a continual subsidance of the more aggravated symptoms.

We now come to consider a different form of fracture, which although in appearance, and form symptoms which may at first present themselves, would not seem to require any very active treatment, yet when understood, demands promptness, as well as operatial interference.

I have reference to what is termed *punctured fracture*, where a sharp body impinges on and penetrates the cranium. The wound in both the external and internal tables, may be no greater than the object which has produced it. But like a nail that has been driven through a broad, its entrance may be smooth, yet when seen upon the other side, it has its spiculæ presenting inwards, which may still remain attached—or may have been entirely separated. If the former, the symptoms of injury are soon present, the spiculæ having penetrated the membranes and perhaps injured the brain itself; but if the latter, the detached portions will only remain in contact with the dura mater—then losing vitality must act as a foreign substance, and if not removed, must inevitably, sooner or later, destroy life.

Two cases of this form of fracture have come under my own eye within the last six weeks. During the month of April, I was called with Prof. Knowles, to see a lad of 16 years, who had been thrown from his horse, his head striking a small stump, producing a punctured fracture near the angle of union of the frontal and parietal bones with the sphenoid of the right side. We found the patient in a comatose condition arising evidently from compression, either in consequence of depressed bone—or extravasated blood—or from both. Finding that operative interference was immediately demanded, I proceeded to operate. The scalp was wounded but slightly; after dissecting this away, we found the opening in the external table still less—but proceeded to use the trephine until the portion it included was removed. The internal table was considerably fractured and depressed; after the removal of all the fragments which amounted to seven, the symptoms of compression did not subside. After a close examination, we found the dura mater somewhat pale and distended, with a feeling of fluctuation. I immediately made an incision through the dura mater, from which escaped from one to two ounces of blood. Immediately after its removal, the patient revived and for several hours presented the most encouraging symptoms of recovery, but in vain. The extent of injury, as was at first suspected, and afterwards proved, extended to the base of the brain, when from the compression, the case was terminated in some 15 hours.

The symptoms in this case were sufficiently plain to demand immediate action; but had not extravasation existed, a question might arise as to the propriety of operative interference.

I will now refer to another case in which the symptoms differ very materially from the one just mentioned, yet demanding equally prompt interference.

A child of Mr. Gamble, a respectable citizen of Montrose, in this county, aged about 20 months, was standing upon a chair—losing its balance it fell, its head coming in contact with a broken tumble, which had fallen from its hand upon the floor—driving an angular piece of the thickest portion of the glass into the frontal bone, near its connection with the right parietal. Dr. Sears was immediately called, but by every effort could not remove the penetrating fragment. I was called to see the case with the Doctor, as well as Drs. Anderson and Slonaker of the same place. It was one of true punctured fracture, the extent of which could not be accurately determined. The little sufferer did not present at the time we saw him—which was several hours after the accident—any symptoms which would tend to alarm the careless spectator. He was somewhat restless—some acceleration of the pulse, the eye expressive of irritability, but no very strong symptoms of compression. After a careful examination and consultation in the case, we were united in the adoption of the proper course.

I proceeded to trephine, after having made the necessary dissection of the scalp, using a small sized instrument, cutting near to the line of puncture in the external table until the diplœ and internal table were reached. At this point we found the glass could be broken down and much of it removed. After a careful examination into the condition of the internal table, we found it punctured, but not to the extent of the external. I then abandoned the trephine and resorted to the use of Hays' saw, removing an angle of the external table which had been surrounded by the trephine, giving an opportunity for removing the small depressed portion of the internal. This was resorted to in preference to a removal of the entire piece around which the trephine had played. Before resorting to the saw to complete the removal of the affected part, a question might arise as to the propriety of allowing the external table which had been cut through, and from which the pericranium had been separated, to remain. This I looked upon as the safer plan, knowing that to remove both tables would be to endanger, to a greater or less extent, the membranes of the brain: while the close connection of the vitality of the internal with that of the external, would likely vitalize it and prevent exfoliation, which would inevitably follow were it to die.

The result in this case has been favorable, the patient having entirely recovered without any unpleasant symptoms.

Punctured fracture therefore differs from other forms of fracture of the cranium in demanding operative interference, whether head symptoms are or are not present. These are sure to supervene upon this form of fracture, sooner or later, while by early interference we may remove the exciting cause, and prevent all inflammatory action.

